# The role of attention bias malleability in experiencing pain and associated disability

**DOI:** 10.7717/peerj.17430

**Published:** 2024-06-03

**Authors:** Justine L. Mac Goris, Jemma Todd, Patrick J.F. Clarke, Alicia M. Hughes, Claus Vögele, Dimitri M.L. Van Ryckeghem

**Affiliations:** 1 Department of Clinical Psychological Science, University of Maastricht, Maastricht, Netherlands; 2 School Psychology, University of Sydney, Sydney, Australia; 3 Curtin School of Population Health, Faculty of Health Sciences, Curtin University of Technology, Curtin, Australia; 4 Department of Psychological Medecine, King’s College London, University of London, London, United Kingdom; 5 Department of Behavioral and Cognitive Sciences, University of Luxembourg, Esch-sur-Alzette, Luxembourg; 6 Department of Experimental-Clinical and Health Psychology, Ghent University, Ghent, Belgium

**Keywords:** Chronic pain, Attentional bias, Attentional bias malleability, Attentional bias modificiation

## Abstract

**Background:**

Attentional processing of pain has been theorized to play a key role in the severity of pain and associated disability. In particular attentional bias towards pain information, resulting in poor pain outcomes, has been extensively researched. Recently, the idea was put forward that attention bias malleability (AM), *i.e.*, the readiness to acquire an attentional bias irrespective of its direction, may be key in predicting poor pain outcomes. We tested this hypothesis in two studies.

**Methods:**

In Study 1, 55 healthy participants completed an AM paradigm, followed by an experimental heat pain paradigm probing pain experience and pain-related task interference. In Study 2, 71 people with chronic pain completed an AM paradigm and questionnaires probing pain experience and associated disability.

**Results:**

In Study 1, including healthy participants, no relationship was found between AM indices and experimental pain outcomes. In Study 2, including chronic pain patients, results indicated that higher levels of overall AM were related to higher levels of pain experience and disability.

**Conclusion:**

This study partially supports the hypotheses that the degree to which individuals can adapt their attentional preference in line with changing environmental conditions is associated with poor pain outcomes. However, future research is needed to clarify inconsistent findings between healthy volunteers and chronic pain patients as well as to determine the causal status of AM in poor pain outcomes.

## Introduction

Pain is hardwired to draw attention, urging a person to react in order to protect the individual from more injury. Despite its importance for survival (*i.e.,* by alarming the body in order to protect it from further harm when a noxious stimulus is experienced), if chronic, pain can lose its adaptive function, becoming a major health problem (Price & Dussor, 2014). Indeed, when chronic, pain often becomes a false alarm signal interfering with ongoing activities (*e.g.*, work ability, physical, emotional and social functioning) and goal pursuit, causing a significant burden to people experiencing chronic pain and their environment (Gatchel et al., 2007). Contemporary models propose that attentional processing of pain information plays a pivotal role in the development and maintenance of chronic pain (Todd et al., 2018; Van Ryckeghem et al., 2019). In particular, it has been suggested that people who exhibit a heightened focus on pain-related information (*i.e.,* pain-related attention bias) tend to report amplified levels of pain and disability and have a higher likelihood for developing chronic pain see Abudoush et al. (2023) and Van Ryckeghem & Crombez (2018) for an overview of theoretical accounts. Furthermore, numerous experimental studies have shown that the level of pain-related attention bias is increased in chronic pain patients compared to healthy volunteers (Abudoush et al., 2023; Crombez et al., 2013; Todd et al., 2018). Yet, despite these findings, available studies have often failed to show that changes in pain-related attentional bias are key in improving pain outcomes (Todd et al., 2015). This has led some scholars to focus on the degree to which people can adapt their attentional preference in line with changing environmental conditions as a key factor in poor pain outcomes (see also Van Ryckeghem et al., 2019). In particular, it is hypothesized that individual differences in the ability to align (*i.e.,* both in terms of acquiring and relinquishing) patterns of selective attention with changing environmental conditions, labelled ‘attentional bias malleability (AM)’, may influence the experience of pain and related interference with functioning. This notion is in line with findings in the field of anxiety where studies have shown that AM predicts anxiety-related outcomes (Clarke, MacLeod & Shirazee, 2008; Taylor, Bomyea & Amir, 2011; Clarke, Chen & Guastella, 2012). In particular, higher levels of AM have been shown to predict elevated trait anxiety in response to extended stressors (Clarke, MacLeod & Shirazee, 2008) as well as reductions in anxiety following therapy (Clarke, Chen & Guastella, 2012). Pain-related AM may also play a pivotal role in daily pain outcomes. In particular, it can be hypothesized that people who more quickly align patterns of selective attention with changing environmental conditions may render some individuals more vulnerable for developing and maintaining (chronic) pain and disability. That is, if greater pain-related AM results in the acquisition of heightened attentional bias for pain-related information in a context of (continuing threat of) pain, this may in turn contribute to higher levels of task interference/disability and the maintenance of chronic pain.

We performed two studies to investigate to what extent individual differences in pain-related AM (*i.e.,* toward pain information, away from pain information, irrespective of its direction) are associated with well-controlled experimental and clinical pain outcomes. In a first study, we investigated if people’s level of AM for pain information related to experimental pain outcomes (*i.e.,* cold pressor pain experience and related task interference). To do so, an experimental study was performed with 55 healthy participants, who completed an AM assessment task and a cold pressor task (with and without competing tone detection task). In a second study we investigated if people’s level of pain-related AM was associated to clinical pain outcomes. An online study was performed with 71 people with chronic pain who completed a pain-related AM assessment task and filled out a questionnaire battery probing daily levels of pain experience and disability. It was hypothesized that participants’ propensity to adapt attention bias (*i.e.,* either toward pain, away from pain or irrespective of its direction) would predict poor experimental pain outcomes (i.c., decreased pain threshold, increased pain intensity and increased pain-related task interference; Study 1) and clinical pain outcomes (i.c., increased pain severity, increased pain disability; Study 2) above and beyond participants’ baseline level of pain-related attention bias. Finally, associations between individual difference variables, including pain worrying, anxiety and depression, and participants’ level of pain-related AM were investigated because existing theoretical accounts have suggested that these individual difference variables may underly the presence of pain-related attention biases (Eccleston & Crombez, 2007; Schoth, Delgado Nunes & Liossi, 2012; Crombez et al., 2012; Van Ryckeghem et al., 2019; Michaelides & Zis, 2019), and may therefore potentially be associated to pain-related AM.

## Experiment 1

## Method

### Participants

Participants were 55 individuals who were recruited *via* university platforms or social media. Inclusion criteria were being aged between 18 and 60 years, normal or corrected-to-normal vision (*e.g.*,  *via* glasses) and a good understanding of English as the experimental session took place in English. People were excluded if they reported current mental conditions, severe and/ or acute pain complaints or neurological conditions (*e.g.*, epilepsy). Participants were also excluded if they used a drug that could affect the central nervous system or medication impacting on somatosensory sensitivity. Additionally, to ensure safety, participants were excluded if they reported heart problems, an electronic implant or were pregnant. Power calculations indicated that for a linear regression with one primary predictor and three control variables, to detect a medium effect size with 80% power, a sample size of 55 participants was needed. Of the 55 participants who were recruited, two participants had to be removed from the analysis, *i.e.,* because the data of the experiment were not properly registered (*n* = 2). Experimental procedures were approved by the Ethics Review Panel of the University of Luxembourg (ERP 20-079 Malleability), and written informed consent was obtained from all participants. In addition, this study was pre-registered (https://osf.io/edr6k/).

### Experimental tasks and methods

#### Task stimuli of Attention bias malleability paradigm

The AM task stimuli consist of a wordlist containing 72 pain words and 72 neutral words sourced from prior research (Todd et al., 2015; Todd, Sharpe & Colagiuri, 2016; Vermeir et al., 2023), matched for number of letters and syllables (see File S1). Words were randomly divided into three lists of 24-word pairs, whereby word frequency did not significantly differ between pain words and neutral words (Pallier, New & Bourgin, 2019). Each word pair was presented 4 times per assessment block, and 12 times per training block. To ensure that attentional bias assessment was not influenced by word familiarity, the first word list was used for the first assessment and first training block, the second word list was used for the second assessment and second training block, and the third word list was used for the final assessment block. Presentation order of the word lists was counterbalanced across participants.

#### Attention bias malleability paradigm

The AM paradigm was presented *via* Inquisit Millisecond software (Inquisit 5; Seattle, WA, USA: Millisecond Software) on a 60-Hz, 19-inch color monitor. The AM paradigm is based on the traditional dot-probe paradigm (*e.g.*, Todd et al., 2015; Todd, Sharpe & Colagiuri, 2016) assessing attention bias for pain (Clarke, MacLeod & Shirazee, 2008; Clarke, Chen & Guastella, 2012). On each trial of the AM paradigm, individuals were presented with a fixation cross in the center of the screen (500 ms), immediately followed by a vertically aligned word pair in 1-cm high white block text. One word appeared above, while the other appeared below, the location of the fixation cross. The word pair was separated by a distance of three cm, subtending slightly less than a 3° visual angle of separation at 60 cm viewing distance (*i.e.,* the distance between participant and monitor). The word pair remained on screen for 500 ms and was replaced by a probe that appeared in either of the two-word locations. The probe consisted of a ‘<’ or ‘>’ which remained on the screen until response. Participants’ response consisted of a ‘<’ or ‘>’ button press using a QWERTY keyboard. Trials were presented with an inter-trial interval of 500 ms. In addition, an error message (‘Incorrect’) was displayed for 2,000 ms in case of an incorrect response, to increase participants’ accuracy. The AM paradigm consists of 5 blocks: 2 training blocks where contingency favored to selectively attend toward either the pain or neutral stimuli (block 2 and 4) and 3 assessment blocks where no contingency existed between the pain and neutral stimuli (block 1, 3 and 5). Each training block comprised 288 trials where the probe consistently followed the pain word (training towards pain information) or consistently followed the neutral word (training away from pain information), with counterbalanced block order presentation between participants. The assessment blocks consisted of 96 trials in which the probe was equally likely to follow the pain word as the neutral word (48 pain congruent trials, 48 pain incongruent trials). Faster responses to identify probes following pain words compared to probes following neutral words was interpreted as indication of attentional bias towards pain information.

#### Random Interval Repetition (RIR) task

The RIR-task was used to assess pain-related task interference (Verhoeven et al., 2010; Van Ryckeghem, Van Damme & Vervoort, 2018; Van Ryckeghem & Crombez, 2018). In particular, the RIR-task is an attention-demanding tone-detection task, which requires executive processing (Vandierendonck, De Vooght & Van der Goten, 1998). Previous research has shown that performance of the RIR task is reduced by the experience of pain (*e.g.*, Van Ryckeghem, Van Damme & Vervoort, 2018). During this task, participants were required to respond (*i.e.,*  *via* button press) as quickly and correctly as possible to tones (tone duration = 150 ms; tone pitch = 750 Hz; with interval of 900 ms or 1,500 ms (50% each in random order) from onset to onset of two consecutive tones) through headphones (Bose QC35). In this study, the total RIR-task duration was 4 min during which 201 tones were presented. Responses were made by pressing a button pressing device, held in the right hand allowing to perform the RIR task while perceiving heat pain stimuli (see section ‘Heat pain induction’). Task performance was assessed in terms of reaction times (RT) and error rate, which were computed by taking into account anticipations (button press prior to tone) and omissions (absent button presses when a tone was presented) (Vandierendonck, De Vooght & Van der Goten, 1998; Verhoeven et al., 2010). The increase of reaction time duration and error rate during the RIR task with heat pain compared to the RIR task without heat pain were interpreted as indices of pain interference.

#### Heat pain induction

Heat pain stimuli were delivered using the Contact Heat Evoked Potentials Stimulator (CHEPS) of the Medoc Neuro Sensory Analyzer, Model TSA-II (Medoc Ltd. Advanced Medical Systems, Ramat, Yishai, Israel). The thermode was secured with a Velcro strap to the inside of the participant’s wrist of the left hand. For this study, stimuli had a duration of 24 s (2 s increase, 20 s plateau phase, 2 s decrease). For the entire experiment, the baseline temperature of the thermode was 32 °C. Participants’ pain threshold and pain tolerance for these pain stimuli were assessed using a simple staircase procedure. For this staircase, the starting temperature was 38 °C. The temperature was subsequently increased in 0.5 °C steps, and participants being requested to indicate whether the heat stimulus was painful (pain threshold). Once participants indicated that the heat stimulus was painful, participants were asked for the following heat stimuli whether the heat stimulus was of the highest level they could tolerate (tolerance level). Once tolerance level was reached (or 48 °C for safety purposes), the staircase was ended. The interstimulus interval during the staircase was 20 s at baseline temperature. For the assessment of task interference, a stimulus of 43.5 °C was used, except for those where the tolerance level was below this temperature, for whom the tolerance level temperature was used (*n* = 14).

During assessment of task interference, the heat stimulus was presented 5 times, with an interstimulus interval of 24 s, totaling a duration of 240 s (whereby the sequence could start with a 24-second pain period or a 24-second non-pain period).

### Self-report measures

#### Individual differences

*Pain worrying* was assessed using the Pain Catastrophizing Scale (Sullivan, Bishop & Pivik, 1995; Crombez et al., 2020). The PCS is a 13-item self-report questionnaire assessing one’s thoughts and feelings associated with pain-related events and contains three subscales, *i.e.,* Helplessness, Magnification, and Rumination. All items are rated on a 5-point Likert scale ranging from 0 (not at all) to 4 (always). Research has shown that the PCS is a valid and reliable scale in both, healthy and clinical populations (Van Damme et al., 2002).

*Depressive mood, anxiety and stress* were assessed using the Depression Anxiety Stress Scales. The DASS-21 (Lovibond & Lovibond, 1995) consists of three self-report scales designed to measure negative emotional states of depression, anxiety, and stress. Each of the three DASS-21 scales contains seven items. Items are answered on a 4-point Likert scale ranging from 0 (“did not apply to me at all”) to 3 (“applied to me very much, or most of the time”). Scores for depression, anxiety and stress are calculated by summing the scores for the items of the respective scales. The DASS-21 was found to be reliable in clinical and community samples (Antony et al., 1998).

#### Attention to pain

Attention to pain during the RIR task was measured by averaging the score on two items of a self-report questionnaire: “How much attention did you pay to the heat stimuli during the tone-detection task?” and “To which extent could you remove the heat stimulus out of your mind during the tone-detection task?” (Reverse-scored). Participants rated both questions using a 11-point Likert scale (0 = “no attention at all”; 10 = “a lot of attention”) (Verhoeven et al., 2010; Verhoeven et al., 2011; Van Ryckeghem, Van Damme & Vervoort, 2018).

### Procedure

Upon arrival in the laboratory at the University of Luxembourg, participants received information concerning the experiment session and were informed that the aim of the experiment was to investigate how an emotional event influences cognitive functioning. Participants were informed that they would experience heat pain stimuli during the experiment session. Next, participants completed the questionnaires (online *via* Qualtrics), after which the experimenter explained the AM paradigm. Next, participants were asked to put on noise-cancelling headphones for the entire experiment. After completion of the AM paradigm participants performed the heat pain staircase procedure, which was immediately followed by the pain interference assessment for which people performed the RIR task while experiencing alternating blocks of heat pain and no pain (see section 1.2.4). Following the RIR task, participants filled out a final questionnaire probing attention for pain and pain experience during the RIR task. Contrasting the pre-registration, pain experience measures during the RIR task were not included in the analyses as they may be confounded by task performance (*i.e.,* distraction efficacy), which may differ between participants. Afterwards participants were debriefed and thanked for their participation with a 10 EUR voucher or course credits. The duration of the complete experiment was approximately 60 min.

### Data-analysis plan

Before performing analyses, incorrect responses and responses faster than 200 ms or slower than 2,000 ms were removed from the AM data. In addition, for each individual, trials with a response latency >2.5 Median Absolute Deviation (MAD) of the median were removed from the analyses of the assessment blocks, resulting in a small loss of data (*i.e.,* data loss ranging between 7.8% and 9.1% for the assessment blocks, *e.g.*, Todd et al., 2023). Finally, participants were excluded if their accuracy was less than 75%. An attention bias (AB) index was then calculated by subtracting the mean latency of congruent trials from the mean latency from incongruent trials for each assessment block separately (*i.e.,* Blocks 1, 3, 5). An index of AM away from pain information was calculated by subtracting the AB index from the assessment block after training from the assessment block before training attention away from pain information (AM_Away_ index). Similarly, an index of AM towards pain information was calculated by subtracting the AB index from the assessment block after training from the assessment block before training attention towards pain information (AM_Towards_ index). Finally, an overall AM index was computed by adding the absolute value from one’s AM index towards pain information and the absolute value from one’s AM index away from pain information (AM_Overall_ index: —AB2 − AB1— +—AB3 − AB2—). Furthermore, and in line with previous research, data from the RIR task were cleaned before RT analyses (Verhoeven et al., 2010; Verhoeven et al., 2011). In detail, RTs faster than 100 ms (*i.e.,* anticipations), outliers (RTs >3 SD above the individual mean) and omissions were removed from the analyses. In addition, we removed each first and last trial from each Block. In total, 9.37% of the RIR responses were excluded from final RT analyses. The pain interference variables (RTs and error rates) were calculated by subtracting the RTs/errors of the trials during the non-painful episodes from the RTs/errors of the trials during the painful episodes.

The main hypotheses, *i.e.,* the predictive value of pain-related AM upon experimental pain outcomes (pain threshold, pain tolerance and task interference), were tested using regression analyses which were performed separately for each AM index. In all regression analyses, we controlled for gender and baseline AB. Age was not included as a control variable due to large homogeneity in the sample. For all analyses Bonferroni corrections were applied for multiple testing. Finally, to explore the relationship between attention indices (*i.e.,* baseline attention bias, AM indices) and individual difference variables, correlation analyses were performed. All data cleaning was performed with R (version 4.0.3; R Core Team, 2020) and statistical analysis were performed with IBM SPSS 27 (SPSS Inc., Armonk, NY, USA).

## Results

### Descriptive statistics

The mean age of the final sample (*n*= 53) included in the analyses was 23.66 years (*SD*= 3.32; range 18–34 years). The majority of this sample (*n*= 29) was female. Participants reported no elevation in their levels of anxiety (*M* = 7.51, *SD* = 5.57), depression (*M* = 8.08, *SD* = 7.34) or stress (*M* = 11.62, *SD* = 7.25) (see also Table 1).

### Attention bias malleability and attention to pain

Three hierarchical regression analyses were performed to investigate the additional value of each of the AM indices in predicting attention to pain. Assumptions for the hierarchical regression were met. For these analyses, gender and baseline AB were added in a first step (control variables) of the hierarchical regression analyses. Analyses indicated that control variables predicted a significant portion of variance in attention to pain (ΔR^2^ = .15, *F*_(2,50)_ = 4.25, *p* = .02). Gender was a significant predictor of attention to pain, indicating that females showed more attention for the pain stimulus than males (*b* = .37, *t*_(52)_ = 2.84, *p* = .01, *f*_2_ = .17). Contrasting our hypotheses, no additional value was found for AM_Towards_ (ΔR^2^ = .01, *F*_(1,49)_ = .51, *p* = .48, *f*_2_ = .01) , AM_Away_ (Δ*R*_2_ = .00, *F*_(1,49)_ = .00, *p* = .98, *f*_2_ = .01) and AM_Overall_ (Δ*R*_2_ = .03, *F*_(1,49)_ = 1.72, *p* = .20, *f*_2_ = .03) in predicting attention to pain.

### Attention bias malleability, pain threshold and pain tolerance

Three hierarchical regression analyses were performed to investigate the additional value of each of the AM indices in predicting pain threshold. For these analyses, gender and baseline AB were added in a first step (control variables) of the hierarchical regression analyses. Results indicated that baseline variables predicted significant portion of variance in pain threshold (ΔR^2^ = .14, *F*_(2,50)_ = 4.02, *p* = .02, *f*_2_ = .16). Baseline AB was a significant predictor of pain threshold, indicating that a higher AB at baseline was associated with a higher pain threshold (*b* = .28, t_(52)_ = 2.09, *p* = .04). Contrasting our hypotheses, no additional value was found of AM_Towards_ index (Δ*R*_2_ = .02, *F*_(1,49)_ = .97, *p* = .33, *f*_2_ = .02), AM_Away_ index (Δ*R*_2_ = .00, *F*_(1,49)_ = .02, *p* = .90, *f*_2_ = .02), and the AM_Overall_ index (Δ*R*_2_ = .00, *F*_(1,47)_ = .02, *p* = .89, *f*_2_ = .00) in predicting pain threshold when controlling for multiple testing. In addition, three hierarchical regression analyses were performed to investigate the additional value of each of the AM indices in predicting pain tolerance. Again, gender and baseline AB were added in a first step (control variables) of the hierarchical regression analyses. Included control variables did not predict pain tolerance (Δ*R*_2_ = .03, *F*_(2,50)_ = .81, *p* = .45). In addition, no significant additional value was found for AM_Towards_ index (Δ*R*_2_ = .07, *F*_(1,49)_ = 4.07, *p* = .05, *f*_2_ = .08), AM_Away_ index (Δ*R*_2_ = .01, *F*_(1,49)_ =.34, *p* = .56, *f*_2_ = .02), and the AM_Overall_ index (Δ*R*_2_ = .02, *F*_(1,49)_ = .91, *p* = .34, *f*_2_ = .03) in predicting pain tolerance when controlling for multiple testing.

**Table 1:** Correlations between attention indices and individual difference variables of Study 1.

		PCS	DASS-A	DASS-D	DASS-S
	Mean (SD)	12.45 (10.73)	7.51 (5.57)	8.08 (7.34)	11.62 (7.25)
AB_Baseline_	−.73 (16.79)	.15	.15	.92	.17
AM_Away_	−3.18 (22.99)	.06	.08	−.23	.01
AM_Towards_	1.69 (28.79)	−.11	−.16	.09	−.04
AM_Overall_	39.51 (28.41)	−.28^*^	.08	.25	−.13

**Notes.**

* *p* < .05.

** *p* < .01.

### Attention bias malleability and pain interference

Six hierarchical regression analyses were performed to investigate the additional value of each of the AM indices in predicting pain interference on RIR task performance (*i.e.,* mean latency, % errors). For all analyses, gender and baseline AB were added in a first step (control variables) of the hierarchical regression analyses. Included control variables didn’t significantly explain variance in the mean latency (Δ*R*_2_ = .09, *F*_(2,50)_ = 2.49, *p* = .09), and % of errors (Δ*R*_2_ = .01, *F*_(2,50)_ = .15, *p* = .86). Next, regression analysis, controlling for multiple testing, including AM_Towards_ as the independent variable indicated no additional value of AM_Towards_ in predicting pain interference on the mean latency (ΔR^2^ = .01, *F*_(1,49)_ = .32, *p* = .57, *f*_2_ = *.01)* and % errors (Δ*R*_2_ = .00, *F*_(1,49)_ = .01, *p* = .94, *f*_2_ = *.00)* during the RIR task. Similarly, regression analysis including AM_Away_ as the independent variable explained no additional variance in predicting pain interference on the mean latency (Δ*R*_2_ = .01, *F*_(1,49)_ = .26, *p* = .61, *f*_2_ =*.01)* and % errors (Δ*R*_2_ = .00, *F*_(1,49)_ = .06, *p* = .81, *f*_2_ =* .01)* of the RIR task. Finally, no additional value of AM_Overall_ was found in predicting pain interference on the mean latency (Δ*R*_2_ = .00, *F*_(1,49)_ = .06, *p* = .81, *f*_2_ =* .01)* and % errors (Δ*R*_2_ = .01, *F*_(1,49)_ = .33, *p* = .57, *f*_2_ =* .01)* of the RIR task.

### Individual differences

In an exploratory step, we investigated whether pain-related attention indices, including baseline AB and AM indices, were related to individual difference variables (see Table 1). Correlation analyses showed that the AM_Overall_ was negatively associated with the PCS, such that individuals with increased levels of AM showed lower levels of pain worrying.

## Experiment 2

### Method

### Participants

Participants were 100 individuals recruited *via* prolific (https://www.prolific.com/), a recruitment application to attract participants for online studies (Peer et al., 2017). Inclusion criteria were being aged over 18 years, suffer from chronic pain and experience of persistent pain for at least three months, being fluent in understanding English and have access to a computer. The study procedure was approved by the Ethics Committee of the University of Sydney, and informed consent was obtained from all participants. This study was pre-registered (https://osf.io/st8e2/). Power calculations indicated that for a linear regression with one primary predictor and three control variables, to detect a medium effect size with 80% power, a sample size of at least 55 participants was required. Due to the online nature of the study, and potentially increased drop-out rate, we did substantially overrecruit participants in our sample. The final sample included 71 participants, after excluding those who indicated that they had not been experiencing pain for the past 3 months (*n* = 22), those who did not complete all aspects of the study (*n* = 6), and those who had low accuracy rates on the AM paradigm (*i.e.,* accuracy < 75%; *n* = 1).

### Experimental tasks and methods

#### AM paradigm

The stimuli and task features of the AM paradigm were identical as in Study 1.

### Self-report measures

Similar to Study 1, participants filled out the PCS (Sullivan, Bishop & Pivik, 1995) and the DASS (Antony et al., 1998; Colster et al., 2008). Additionally, participants completed the Graded Chronic Pain Scale (Von Korff et al., 1992) to assess daily levels of pain experience and disability. The GCPS consists of seven items. Pain intensity items assess pain intensity right now, worst pain intensity over the last six months, and average pain intensity over the last six months. These three items are averaged in a pain intensity score (range 0–100). Pain disability items assess limitation in daily activities because of pain, limitation in recreational, social, and family activities within the last six months, and limitation in the ability to work because of pain within the last six months. These three items are averaged in a pain disability score (range 0–100). Finally, the number of days disabled within the last six months is assessed. All items, except for the number of days disabled, are scored on an 11-point scale (0–10). Previous research has shown that the GCPS is reliable and valid for assessing pain intensity and pain disability in chronic pain samples (Smith et al., 1997).

### Procedure

Via the prolific platform, participants received a link to an online survey programmed in Inquisit (Inquisit 5; Seattle, WA, USA: Millisecond Software) and presented *via* the Inquisit web application. Participants were instructed to sit in a quiet environment during the study (*i.e.,* a room where they do not get disturbed by others), before the online survey started. Participants were presented with an online information statement and gave informed consent by clicking a button on the screen prior to participating. Following consent, participants completed the questionnaire battery. At the end of the survey, participants performed the AM paradigm. The entire duration of the experiment was approximately 45 min, and participants were reimbursed £3.75 for their time.

### Data-analysis plan

Before the analyses were performed on the data of the AM paradigm, incorrect responses, and responses faster than 200ms or slower than 2000ms were removed from the data. In addition, for each individual, trials with a response latency >2.5 MAD of the median were removed from the analyses of the assessment blocks, resulting in a small loss of data (*i.e.,* data loss ranging between 7.9% and 10.8% for the assessment blocks). Similar to Study 1, a baseline AB index and AM indices (AM_Away_ index, AM_Towards_ index, AM_Overall_ index) were calculated. To investigate the relationship between pain-related AM and chronic pain outcomes, separate regression analyses were performed for each AM index for both pain intensity and pain disability scores of the GCPS. In all regression analyses, we controlled for gender, age and baseline AB. Data cleaning was performed with R (version 4.0.3; R Core Team, 2020) and statistical analyses were performed with IBM SPSS 27. For all analyses Bonferroni corrections were applied for multiple testing. Finally, to explore the relationship between attention indices (*i.e.,* baseline AB, AM indices) and individual difference variables, correlation analyses were performed.

## Results

### Descriptive statistics

The final sample comprised 71 people reporting chronic pain that had persisted for at least 3 months. Participants reported experiencing pain for an average of 10.18 years (*SD* = 9.93; range 3 months–39 years). The majority of the sample (*i.e.,* 43 participants) was female, with a wide age range (*M*_age_ = 41.55 years; *SD* = 14.83; range 20–74 years). Participants reported moderate levels of pain intensity (*M* = 51.36; *SD* = 17.51) and had a pain disability score of 41.92 (*SD* = 25.38) on the GCPS. Furthermore, participants reported mild to moderate levels of anxiety (*M* = 10.14, *SD* = 8.93), depression (*M* = 15.75, *SD* = 11.62) and stress (*M* = 17.13, *SD* = 10.64) (see also Table 2).

**Table 2:** Correlations between attention indices and individual difference variables of Study 2.

		PCS	DASS-A	DASS-D	DASS-S
	Mean (SD)	18.07 (13.11)	10.14 (8.93)	15.75 (11.62)	17.13 (10.64)
ABB_aseline_	−.92 (16.02)	−.090	−.010	−.007	.067
AM_Away_	−4.31 (27.58)	−.033	.074	.167	.044
AM_Towards_	5.43 (28.54)	.145	−.022	−.206	−.108
AM_Overall_	43.09 (31.68)	−.050	.042	−.013	−.005

**Notes.**

* *p* < .05.

** *p* < .01.

### Attention bias malleability, pain intensity and pain disability

Three hierarchical regression analyses were performed to investigate the additional value of each of the pain-related AM indices in predicting levels of pain intensity. For all analyses, gender, age and baseline AB were added in a first step (control variables) of the hierarchical regression analyses. Included control variables did not significantly explain variance in pain intensity (Δ*R2* = .06, *F*_(3,67)_ = 1.39, *p* = .26). Furthermore, the results indicated no additional value of AM_Towards_ (Δ*R2* = .00, *F*_(1,66)_ = .00, *p* = .97, *f*_2_ = .01), AM_Away_ (Δ*R2* = .01, *F*_(1,66)_ = 0.74, *p* = .39, *f*_2_ = .01), and AM_Overall_ (Δ*R2* = .05, *F*_(1,66)_ = 4.03, *p* =.05, *f*_2_ = .06) indices in explaining the variance in participants’ pain intensity score when controlling for multiple testing.

Next, three hierarchical regression analyses (controlling for multiple testing) were performed to investigate the additional value of each of the pain-related AM indices in predicting pain disability. Again, for all analyses, gender, age and baseline AB were added in a first step (control variables) of the hierarchical regression analyses. Included control variables did not significantly predict pain disability (Δ*R2* = .06, *F*_(3,67)_ = 1.39, *p* = .25). Furthermore, no additional explanatory value was found for AM_Towards_ (Δ*R2* = .01, *F*_(1,66)_ = .46, *p* = .50, *f*_2_ = .01) and AM_Away_ (Δ*R2* = .01, *F*_(1,66)_ = .95, *p* = .33, *f*_2_ = .01) indices. Yet, the results indicated that the AM_Overall_ index significantly added value in explaining the variance of participants’ level of pain disability (*b* = .27, *t*_(70)_ = 2.59, *p* = .01; Δ*R2* = .10, *F*_(1,66)_ = 7.56, *p* = .01, *f*_2_ =.09), such that increased levels of pain related AM_Overall_ was associated with higher pain disability.

#### Individual differences

To explore whether pain-related attention indices were related to individual difference variables (*i.e.,* pain worrying, depression, anxiety, and stress), correlations analyses were performed. Correlations between the respective variables are listed in Table 2. Yet, no significant correlations were found.

### General discussion

The aim of the present series of studies was to investigate to what extent individual differences in the readiness to acquire an attentional bias for pain are associated with pain and pain disability or pain-related task interference. This was investigated in a controlled experimental condition with healthy volunteers and using self-reported pain and pain disability measures in chronic pain patients. In Study 1, including healthy volunteers, findings did not show an association between pain-related AM indices, heat pain experience and pain-related task interference. These null findings are in contrast with our hypotheses. However, for Study 2, including chronic pain patients, results indicated that increased levels of AM_Overall_ did relate to increased levels of pain disability. As such, findings of current research partially supported the hypotheses that malleability in pain-related attention bias (irrespective of its direction) is associated with pain outcomes.

The finding that higher levels of pain-related AM are associated with higher levels of disability, above and beyond participants’ level of pain-related AB, aligns with previous research in the context of anxiety. In particular, findings of Clarke, Chen & Guastella (2012) suggested that the level of AM is a good predictor of self-reported anxiety, whereby increased readiness to acquire an attentional bias (irrespective of its direction) predicted elevations in trait anxiety in response to extended stress (Clarke, MacLeod & Shirazee, 2008). Based upon these findings, they suggested that variability in the ease with which an attention bias can be acquired (as assessed with the AM paradigm), would predict who will naturalistically develop an attentional preference for threat when exposed to extended stressors. Similarly, Lester et al. (2019) stated that their findings indicate that a stronger preparedness to acquire a threat bias in response to an experimental contingency supporting selective threat processing might forecast the development of an attention bias toward threat when facing an extended stressor, predicting experience of increased levels of anxiety. In corroboration with these suggestions, we propose that current study results may be explained by the fact that high levels of AM for pain-related information facilitate the naturalistic acquisition of an attentional preference for pain-relevant information when exposed to prolonged pain, whereby this attentional preference for pain-relevant information may then fuel the maintenance and exacerbation of chronic pain-related disability. Notably, although not surviving multiple testing correction, findings in chronic pain patients concerning the relationship between pain-related AM and pain experience mirrored the findings between pain-related AM and pain-related disability.

Available research in the anxiety domain does suggests that this is only part of the story, indicating that the (mal-)adaptive value of AM is dependent upon contextual features. Indeed, besides being related to elevated levels of trait anxiety in the context of extended stressors (Clarke, MacLeod & Shirazee, 2008), increased levels of AM were also found to be associated with improvement in psychological therapy outcomes when aiming to positively modify attention biases in therapy (Clarke, Chen & Guastella, 2012) . The impact of context may also explain null findings in our healthy population (Study 1) where, in contrast to Study 2 (chronic pain population), a context of prolonged pain is absent. Together, this suggest that the influence of pain-related AM depends upon an individual’s current environmental contingencies whereby uncontrollable extended stressors, such as chronic pain, render an individual more vulnerable, whereas the introduction of psychological treatment may result in a more favorable outcome due to greater malleability of cognitive processes surrounding pain. Following this reasoning, it could be expected that also in people suffering pain that is relatively manageable, increased levels of AM may result in more favorable outcomes (Todd et al., 2023). This contextual view on AM is in line with contemporary views on the existence and potential impact of attention bias upon pain outcomes (*e.g.*, Van Ryckeghem et al., 2019), which suggest that attention bias is dynamic in nature, with the alignment between cognitive processing of pain-related information with context and goal pursuit, rather than cognitive biases themselves being key for maladaptive outcomes (see also Verhoeven et al., 2010). Hereby it may be essential that valuable goals are identified and activated, so the pursuit of goals become salient (Schrooten & Vlayen, 2010; Schrooten, Vlayen & Morley, 2012; Crombez et al., 2016; Van Damme et al., 2010). Moreover, it has been proposed that pursuing an “important” goal for the individual may increase their attention and inhibit less important goals (Schrooten & Vlayen, 2010; Van Ryckeghem & Crombez, 2018). In clinical practice it may accordingly be of importance to identify salient non-pain related goals (*e.g.*, enjoy hobbies; see also Van Ryckeghem & Crombez, 2018; Van Ryckeghem et al., 2019).

Within the present series of studies, we did not only investigate the role of pain-related AM, but also provided a methodology to assess the readiness to acquire an attention bias for pain-related information. This methodology is informed by available research on anxiety-related disorders (Clarke, Chen & Guastella, 2012), but was also significantly improved upon, as the link between the AM indices and pain outcomes cannot be due to the attention bias modification training itself. In doing so, this study provides a tool that can be used in future research and, in later stages clinical practice, to provide an index of peoples’ readiness to acquire an attentional bias towards or away from pain-related information. Taking this AM index into account may then allow to (a) further interrogate the value of AM in predicting the development and/or maintenance of chronic pain problems and associated disability levels, and (b) increase treatment efficacy by tailoring interventions based upon peoples’ readiness to acquire an AB for pain-related information. At current, only a few studies, predominantly those where attention bias training successfully reduced pain-related attention, reported better pain outcomes following attention bias training (Todd et al., 2015; Sharpe et al., 2012, but see Carleton et al., 2020). Therefore, researchers have aimed to improve traditional attention bias modification paradigms by increasing the relevance of pain-related information (Notebaert et al., 2015; Van Ryckeghem, Van Damme & Vervoort, 2018; Vermeir et al., 2023) or including game features to increase motivation and engagement (Vermeir et al., 2022). Yet, current research suggests that differences in peoples’ readiness to acquire an attentional bias for pain-related information may also, at least partly, explain inconsistent findings in existing literature concerning the effect of attention bias modification to reduce pain and/or disability. Depending upon the proportion of individuals having high levels of AM included in the sample, attention bias modification may be less or more successful in modifying attention biases for pain information. This may explain why some studies found changes in individuals’ level of attention bias for pain-related information, whereas others did not and consequently did not find an impact upon pain outcomes (Van Ryckeghem, Van Damme & Vervoort, 2018; Van Ryckeghem & Crombez, 2018).

Some aspects of the current study require further consideration. First, to assess pain interference in healthy volunteers, we made use of the Medoc to induce heat pain stimuli of 20 s combined with the RIR task performance. The use of multiple stimuli of 20 s differs from previous studies aiming to assess pain interference using the RIR task and pain experience or disability in daily life contexts. It may be that participants are able to elevate their effort for a short duration during which pain is experienced, shielding task performance from interruption, which would not be possible for the longer pain stimuli (*e.g.*, induced *via* the cold pressor task or daily life) (Verhoeven et al., 2010; Verhoeven et al., 2011). Second, participants in Study 1 were mainly pain-free undergraduate students experiencing experimental pain. The homogeneity of the study sample may have limited the variability in individual difference variables and AM, reducing the possibility to find associations with these variables. Therefore, future research may want to replicate the current study in more heterogeneous populations (*e.g.*, non-student populations, older population) to address the impact of individual difference variables upon AM. Third, English proficiency of participants in Study 1 was not explicitly tested but based upon self-evaluation and the observation of the test leader. To ensure that participants have the ability to process semantic and emotional content in an automatic manner future studies using this design may only want to include native English speaking participants. Fourth, within current study, we assessed pain intensity and pain disability in chronic pain patients using self-report measures reflecting over the past six months. Although, we used well-validated measures, they do not allow to assess daily fluctuations in pain intensity and pain disability. More fine-grained assessment methods, such as ecological momentary assessment methodology, do allow to also investigate the role of pain-related AM in daily fluctuations of pain and disability (Van Ryckeghem et al., 2013; Todd et al., 2023). Sixth, it should be noted that attention bias was assessed using the traditional operationalization, *i.e.,* using symbolic information presented in a reaction time paradigm. The paradigm developed to assess AM has similar limitations as the traditional dot-probe paradigm (*i.e.,* the use of static stimuli, low reliability; Dear et al., 2011a; Dear et al., 2011b). Future research should aim to overcome these limitations by using eye-tracking methodology (Franklin, Holmes & Fowler, 2019; Jackson, Yang & Su, 2019), or paradigms that make use of more ecological valid pain stimuli, such as conditioned pain cues (Van Ryckeghem & Crombez, 2018) or realistic pain information presented *via* virtual reality methodology.

## Conclusion

Despite these limitations, the current studies, combining an experimental study in healthy participants and an online study in chronic pain patients, provides important insights in the malleability of attention bias for pain information and its links with pain outcomes. Particularly, the link between pain-related AM, pain and disability in chronic pain patients may provide relevant contributions for research and clinical practice. Current findings do need further replication and consideration in future research.

##  Supplemental Information

10.7717/peerj.17430/supp-1 Supplemental Information 1 Code for attentional bias indices The code for cleaning and creating the attentional bias index to use for the hierarchical linear regression

10.7717/peerj.17430/supp-2 Supplemental Information 2 Code for the self report questionnaires and demographics The code to retrieve all the self report answer to the battery of questionnaire every participant had to take

10.7717/peerj.17430/supp-3 Supplemental Information 3 Code for the RIR task This is the code for the RIR task, cleaning and obtaining the pain interference index and percentage of errors

10.7717/peerj.17430/supp-4 Supplemental Information 4 Every linear regression coefficient for the different models

10.7717/peerj.17430/supp-5 Supplemental Information 5 Syntax for the Linear regression Syntax SPSS file for study 1 and study 2 for the linear regression

10.7717/peerj.17430/supp-6 Supplemental Information 6 The raw data for the study 1 (first part) of the malleability study

10.7717/peerj.17430/supp-7 Supplemental Information 7 The raw data of study 2 (second part) of the malleability study

10.7717/peerj.17430/supp-8 File S1 All the words in the attentional bias task

## Additional Information and Declarations

### Data Availability

The following information was supplied regarding data availability: The code and raw data are available in the Supplemental Files.
