# Supplementary material for: The role of attention bias malleability in experiencing pain and associated disability
Source: PeerJ. 2024 Jun 3;12:e17430. doi: 10.7717/peerj.17430 (PMC11155670; doi:10.7717/peerj.17430)
Supplement: Supplemental Information 4 [file peerj-12-17430-s004.docx]

Supplementary file.

Coefficient of the hierarchical regression.

Study 1.

Table 1. Coefficient for the linear regression with AB baseline, gender, AMtoward, AMaway and AM overall and Attention to pain as the variable of interest.

| Predictors | B | SD | β | t | p-value |
| --- | --- | --- | --- | --- | --- |
| AB pre | .01 | .02 | .05 | .36 | .72 |
| Sex | **1.98** | **.70** | **.37** | **2.84** | **.01** |

*ΔR^2^ = .15, p= .02*

| Predictors | B | SD | β | t | p-value |
| --- | --- | --- | --- | --- | --- |
| AB pre | .01 | .02 | .07 | .53 | .60 |
| Sex | **1.96** | **.70** | **.37** | **2.80** | **.01** |
| AMtoward | .01 | .01 | .10 | .71 | .48 |

*ΔR^2^ = .01, p = .48*

| Predictors | B | SD | β | t | p-value |
| --- | --- | --- | --- | --- | --- |
| AB pre | .01 | .02 | .05 | .36 | .72 |
| Sex | **1.98** | **.70** | **.37** | **2.81** | **.01** |
| AMaway | .00 | .02 | .00 | .03 | .99 |

*ΔR^2^ = .00, p = .98*

| Predictors | B | SD | β | t | p-value |
| --- | --- | --- | --- | --- | --- |
| AB pre | .01 | .02 | .06 | .49 | .63 |
| Sex | **2.00** | **.69** | **.38** | **2.88** | **.01** |
| AMoverall | -.02 | .01 | -.17 | -1.31 | .20 |

*ΔR^2^ = .03, p = .20*

Table 2. Coefficient for the linear regression with AB baseline, gender as the control variable and, AMtoward, AMaway and AM overall as the independent variables and Pain threshold as the variable of interest

| Predictors | B | SD | β | t | p-value |
| --- | --- | --- | --- | --- | --- |
| AB pre | **.03** | **.02** | **.28** | **2.09** | **.04** |
| Sex | .86 | .51 | .22 | 1.69 | .10 |

*ΔR^2^* **= .14; p = .02**

| Predictors | B | SD | β | t | p-value |
| --- | --- | --- | --- | --- | --- |
| AB pre | **.04** | **.02** | **.31** | **2.27** | **.03** |
| Sex | .85 | .51 | .22 | 1.66 | .10 |
| AMtoward | .01 | .01 | .13 | .98 | .33 |

*ΔR^2^* = .02; p= .33

| Predictors | B | SD | β | t | p-value |
| --- | --- | --- | --- | --- | --- |
| AB pre | **.03** | **.02** | **.28** | **2.07** | **.04** |
| Sex | .86 | .52 | .22 | 1.68 | .10 |
| AMaway | .00 | .01 | .02 | .12 | .90 |

*ΔR^2^ = .00,* p=.90

| Predictors | B | SD | β | t | p-value |
| --- | --- | --- | --- | --- | --- |
| AB pre | **.03** | **.02** | **.27** | **2.04** | **.05** |
| Sex | .86 | .52 | .22 | 1.67 | .10 |
| AMoverall | .00 | .01 | .02 | .14 | .89 |

*ΔR^2^= .00*; p= .89

Table 3. Coefficient for the linear regression with AB baseline, gender as the control variables, AMtoward, AMaway , and AMoverall as the independent variables and Pain tolerance as the variable of interest

| Predictors | B | SD | β | t | p-value |
| --- | --- | --- | --- | --- | --- |
| AB pre | .02 | .01 | .18 | 1.26 | .22 |
| Sex | .03 | .41 | .01 | .07 | .95 |

*ΔR^2^* = .03; p= .45

| Predictors | B | SD | β | t | p-value |
| --- | --- | --- | --- | --- | --- |
| AB pre | .02 | .01 | .25 | 1.78 | .08 |
| Sex | .01 | .40 | .00 | .01 | .99 |
| AMtoward | .01 | .01 | .28 | 2.02 | .05 |

*ΔR^2^* = .07; p = .05

| Predictors | B | SD | β | t | p-value |
| --- | --- | --- | --- | --- | --- |
| AB pre | .01 | .01 | .18 | 1.18 | .25 |
| Sex | .02 | .41 | .01 | .05 | .96 |
| AMaway | -.01 | .01 | -.08 | -.58 | .56 |

*ΔR^2^* = .01; p = .56

| Predictors | B | SD | β | t | p-value |
| --- | --- | --- | --- | --- | --- |
| AB pre | .01 | .01 | .16 | 1.16 | .25 |
| Sex | .02 | .41 | .01 | .05 | .96 |
| AMoverall | .01 | .01 | .13 | .96 | .34 |

*ΔR^2^* = .02; p = .3

Table 4. Coefficient for the linear regression with AB baseline, gender as the control variable, AMtoward, AMaway and AM overall as the independent variables and Pain interference (mean latency) as the variable of interest.

| Predictors | B | SD | β | t | p-value |
| --- | --- | --- | --- | --- | --- |
| AB pre | **.43** | **.20** | **.30** | **2.20** | **.03** |
| Sex | -3.82 | 6.53 | -.08 | -.58 | .56 |

*ΔR^2^* = .09; p = .09

| Predictors | B | SD | β | t | p-value |
| --- | --- | --- | --- | --- | --- |
| AB pre | .40 | .21 | .28 | 1.96 | .06 |
| Sex | -3.71 | 6.58 | -.08 | -.57 | .58 |
| AMtoward | -.07 | .12 | -.08 | -.57 | .57 |

*ΔR^2^* = .01; p =.57

| Predictors | B | SD | β | t | p-value |
| --- | --- | --- | --- | --- | --- |
| AB pre | **.44** | **.20** | **.31** | **2.23** | **.03** |
| Sex | -3.72 | 6.58 | -.08 | -.57 | .58 |
| AMaway | -.07 | .15 | -.07 | .51 | .61 |

*ΔR^2^* = .01; p = .61

| Predictors | B | SD | β | t | p-value |
| --- | --- | --- | --- | --- | --- |
| AB pre | **.44** | **.20** | **.30** | **2.20** | **.03** |
| Sex | -3.79 | 6.59 | -.08 | -.58 | .57 |
| AMoverall | -.03 | .12 | -.03 | -.25 | .81 |

*ΔR^2^* = .00; p = .81

Table 5. Coefficient for the linear regression with AB baseline, gender as the control variables and, AMtoward, AMaway and AM overall as the independent variables and Pain interference (% of errors) as the variable of interest.

| Predictors | B | SD | β | t | p-value |
| --- | --- | --- | --- | --- | --- |
| AB pre | -.02 | .07 | -.05 | -.36 | .72 |
| Sex | -.87 | 2.24 | -.06 | -.39 | .70 |

*ΔR^2^* = .01; p = .86

| Predictors | B | SD | β | t | p-value |
| --- | --- | --- | --- | --- | --- |
| AB pre | -.03 | .07 | -.05 | -.36 | .72 |
| Sex | -.86 | 2.26 | -.05 | -.38 | .71 |
| AMtoward | -.00 | .04 | -.01 | -.07 | .94 |

*ΔR^2^* = .00; p = .94

| Predictors | B | SD | β | t | p-value |
| --- | --- | --- | --- | --- | --- |
| AB pre | -.02 | .07 | -.05 | -.36 | .75 |
| Sex | -.85 | 2.26 | -.05 | -.38 | .71 |
| AMaway | .01 | .05 | .03 | .24 | .81 |

*ΔR^2^* = .00; p = .81

| Predictors | B | SD | β | t | p-value |
| --- | --- | --- | --- | --- | --- |
| AB pre | -.03 | .07 | -.06 | -.41 | .69 |
| Sex | -.89 | 2.26 | -.06 | -.39 | .70 |
| AMoverall | .02 | .04 | .08 | .58 | .57 |

*ΔR^2^* = .01; p = .57

Study 2.

Table 1. Coefficient for the linear regression with AB baseline, gender, age as the control variables, and AMtoward, AMaway and AMoverall as the independent variable and Pain intensity as the variable of interest.

| Predictors | B | SD | β | t | p-value |
| --- | --- | --- | --- | --- | --- |
| ABpre | -.01 | .13 | .01 | .06 | .95 |
| Gender | 6.71 | 4.05 | .20 | 1.65 | .10 |
| Age | .16 | .14 | .14 | 1.15 | .27 |

*ΔR^2^* = .06; p = .26

| Predictors | B | SD | β | t | p-value |
| --- | --- | --- | --- | --- | --- |
| ABpre | .01 | .14 | .01 | .07 | .94 |
| Gender | 6.72 | 4.11 | .20 | 1.64 | .11 |
| Age | .16 | .14 | .14 | 1.14 | .26 |
| AMtoward | .00 | .01 | .01 | .04 | .97 |

*ΔR^2^* = .00; p = .97

| Predictors | B | SD | β | t | p-value |
| --- | --- | --- | --- | --- | --- |
| ABpre | -.04 | .14 | -.04 | -.29 | .77 |
| Gender | 6.95 | 4.07 | .20 | 1.71 | .09 |
| Age | .15 | .14 | .12 | 1.03 | .31 |
| AMaway | -.07 | .08 | -.11 | -.86 | .39 |

*ΔR^2^* = .01; p = .39

| Predictors | B | SD | β | t | p-value |
| --- | --- | --- | --- | --- | --- |
| ABpre | .04 | .13 | .04 | .33 | .74 |
| Gender | 5.15 | 4.04 | .15 | 1.28 | .21 |
| Age | .19 | .14 | .16 | 1.39 | .17 |
| AMoverall | **.13** | **.07** | **.24** | **2.01** | **.05** |

*ΔR^2^* = .05; p= .05

Table 2. Coefficient for the linear regression with AB baseline, gender, age as the control variables, and AMtoward, AMaway and AMoverall as the independent variable and Pain disability as the variable of interest.

| Predictors | B | SD | β | t | p-value |
| --- | --- | --- | --- | --- | --- |
| ABpre | -.04 | .19 | -.02 | -.19 | .85 |
| Gender | 9.37 | 5.87 | .19 | 1.60 | .12 |
| Age | .26 | .20 | .15 | 1.27 | .21 |

*ΔR^2^* = .06; p = .25

| Predictors | B | SD | β | t | p-value |
| --- | --- | --- | --- | --- | --- |
| ABpre | .00 | .20 | .00 | -.00 | .99 |
| Gender | 9.82 | 5.94 | .20 | 1.66 | .10 |
| Age | .26 | .21 | .15 | 1.27 | .21 |
| AMtoward | .08 | .11 | .08 | .68 | .50 |

*ΔR^2^* = .01; p = .50

| Predictors | B | SD | β | t | p-value |
| --- | --- | --- | --- | --- | --- |
| ABpre | -.12 | .21 | -.07 | -.57 | .57 |
| Gender | 9.77 | 5.89 | .20 | 1.66 | .10 |
| Age | .23 | .21 | .14 | 1.12 | .26 |
| AMaway | -.12 | .12 | -.13 | -.97 | .33 |

*ΔR^2^* = .01; p = .33

| Predictors | B | SD | β | t | p-value |
| --- | --- | --- | --- | --- | --- |
| ABpre | .03 | .18 | .02 | .17 | .87 |
| Gender | 6.37 | 5.71 | .13 | 1.12 | .27 |
| Age | .32 | .20 | .19 | 1.63 | .11 |
| AMoverall | **.26** | **.09** | **.32** | **2.75** | **.01** |

*ΔR^2^* = .10; p = .01
