# Supplementary material for: The role of attention bias malleability in experiencing pain and associated disability
Source: PeerJ. 2024 Jun 3;12:e17430. doi: 10.7717/peerj.17430 (PMC11155670; doi:10.7717/peerj.17430)
Supplement: File S1 [file peerj-12-17430-s008.pdf]

Supplementary File 1

Word lists

List 1	
**Pain**	**Neutral**
FLICKERING	WATERFALLS
THROBBING	WALLPAPER
SHOOTING	POLISHED
BORING	SWIVEL
DRILLING	WHIRLING
SHARP	PLATE
BURNING	PAINTED
STIFF	SKIRT
TUGGING	REFRESH
PINCHING	POSTMARK
PAIN	ROOM
STABBING	BANISTER
TIRING	PURPLE
EXHAUSTING	VOLLEYBALL
VICIOUS	MORNING
ANNOYING	TOMORROW
MISERABLE	AUTOMATIC
TROUBLESOME	APPRECIATED
UNBEARABLE	RELAXATION
CRUEL	AGAIN
PUNISHING	CHARACTER
DISCOURAGING	OVERSTEPPING
EXCRUCIATING	SIMULTANEITY
SICKENING	MAGAZINES

List 2	
**Pain**	**Neutral**
TENSION	POPCORN
ACHE	SPUN
SPLITTING	CORKSCREW
GNAWING	DAWNING
HURTING	SPEAKER
POUNDING	CUPBOARD
STING	WHISK
SPASM	PIPER
CRAMPING	CRAMMING
SORE	PEGS
SCALDING	WARDROBE
TWITCHING	LIGHTBULB
WRETCHED	FLAWLESS
GRUELLING	PINEAPPLE
SUFFOCATING	UNDEMANDING
BLINDING	KINDLING
INTENSE	NOTHING
DREADFUL	MATTRESS
AGONIZING	ELABORATE
TORTURING	RECYCLING
HORRIBLE	OPPOSITE
KILLING	FASHION
DEBILITATING	BICENTENNIAL
DEVASTATING	ARRANGEMENT

List 3	
**Pain**	**Neutral**
SEARING	LOANING
CLAWING	DRESSER
PULSATING	SIDEBOARD
PENETRATING	PREDICTABLE
PIERCING	BOOKCASE
PRESSING	SWIMMING
BEATING	ADDRESS
CRUSHING	BASEMENT
INFLAMED	ORNAMENT
SQUEEZING	NEIGHBOUR
TEARING	PICTURE
TENDER	DINING
NAGGING	SILENCE
DISTRESSING	FACILITATOR
UNCONTROLLABLE	HYPOTHETICALLY
TERRIBLE	EXCHANGE
DIFFICULT	AMENDMENT
DEPRESSING	FRIENDSHIP
FEARFUL	MESSAGE
SUFFERING	NUTRITION
AGGRAVATING	CONVERTIBLE
UNPLEASANT	FORGETTING
UNCOMFORTABLE	SOPHISTICATED
FRIGHTFUL	HATCHLING
